# Supplementary figures and images for: Identifying clinical skill gaps of healthcare workers using a digital clinical decision support algorithm during outpatient pediatric consultations in primary health centers in Rwanda
Source: PLoS One. 2025 Jun 3;20(6):e0318284. doi: 10.1371/journal.pone.0318284 (PMC12132983; doi:10.1371/journal.pone.0318284)

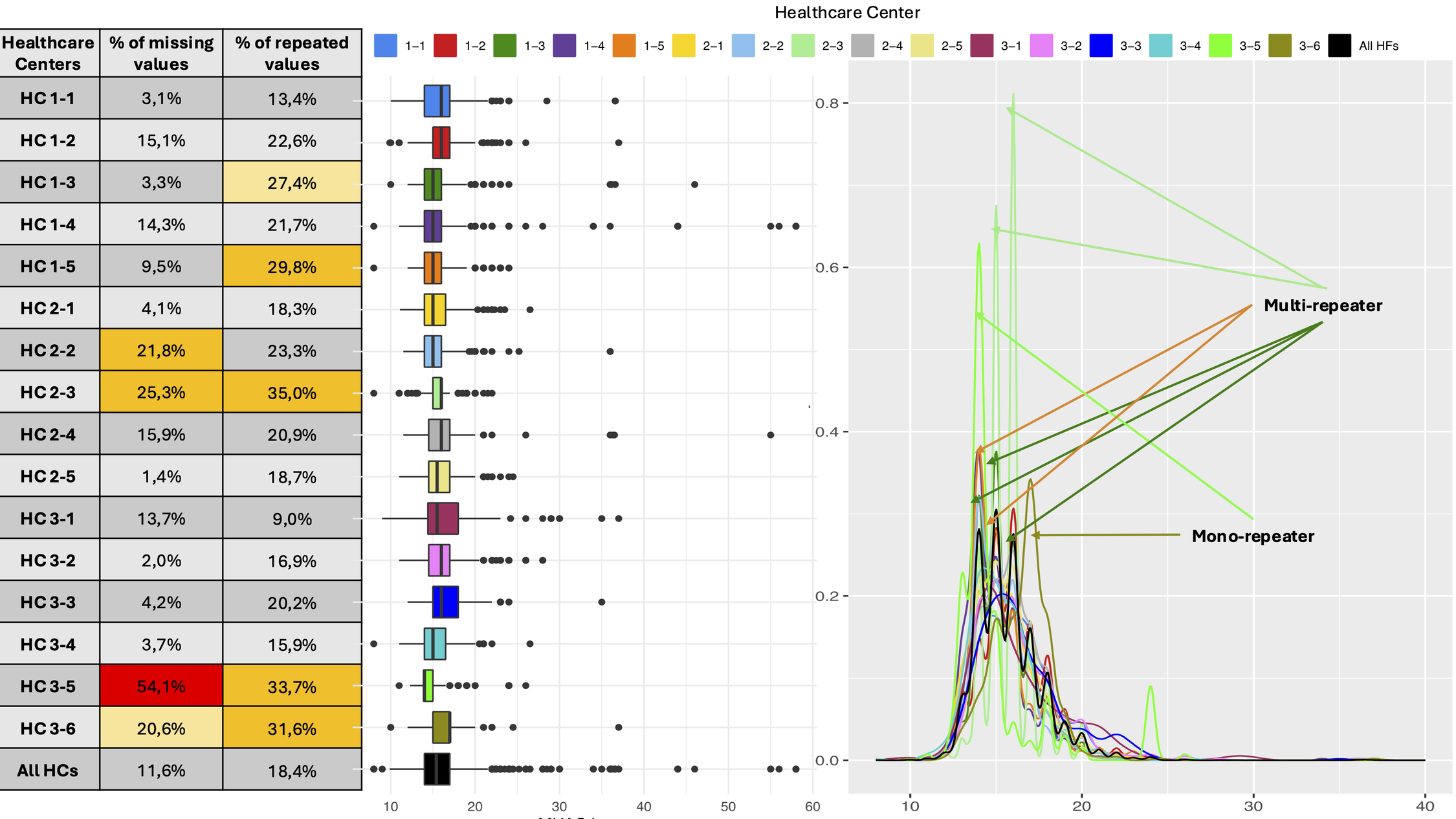

Supplement: S1 Fig — The left table highlights the prevalence of missing and repeated values. The middle boxplot shows distributions of mid-upper arm circumference values by health center, compared to the overall distribution in all health centers (bottom). The right plot shows frequency distributions by health center. This figure links to Table 3, which identifies the following patterns: 1) HC 3–6 as a mild, HCs 2–2 and 2–3 as moderate and HC 3–5 as severe skippers; HC 3–5 and HC 3–6 as a moderate mono-repeater; HC 1–3 as a mild and HCs 1–5 and 2–3 as moderate muliti-repeaters. (TIF) [file pone.0318284.s001.tif]

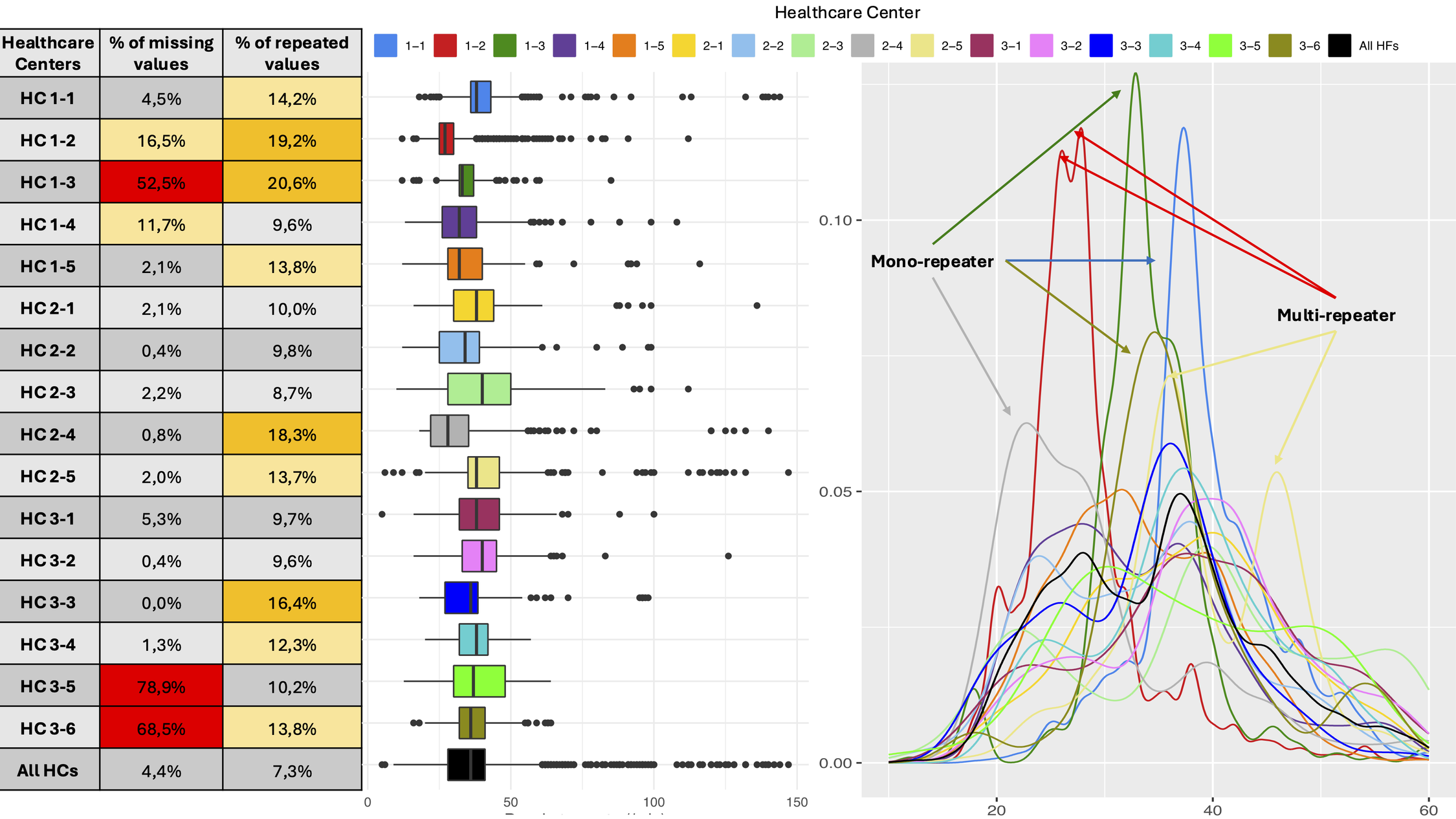

Supplement: S2 Fig — The left table highlights the prevalence of missing and repeated values. The middle boxplot shows distributions of respiratory rate values by health center, compared to the overall distribution in all health centers (bottom). The right plot shows frequency distributions by health center. This figure links to Table 3, which identifies the following patterns: 1) HC 1–2 and 1–4 as a mild, and HC 1–3, 3–5 and 3–6 as severe skippers; HC 1–1, 1–5, 3–4 and 3–6 as mild, HC 1–3, 2–4 and 3–3 as a moderate mono-repeater; HC 2–5 as a mild and HCs 1–2 as moderate multi-repeaters. (TIF) [file pone.0318284.s002.tif]

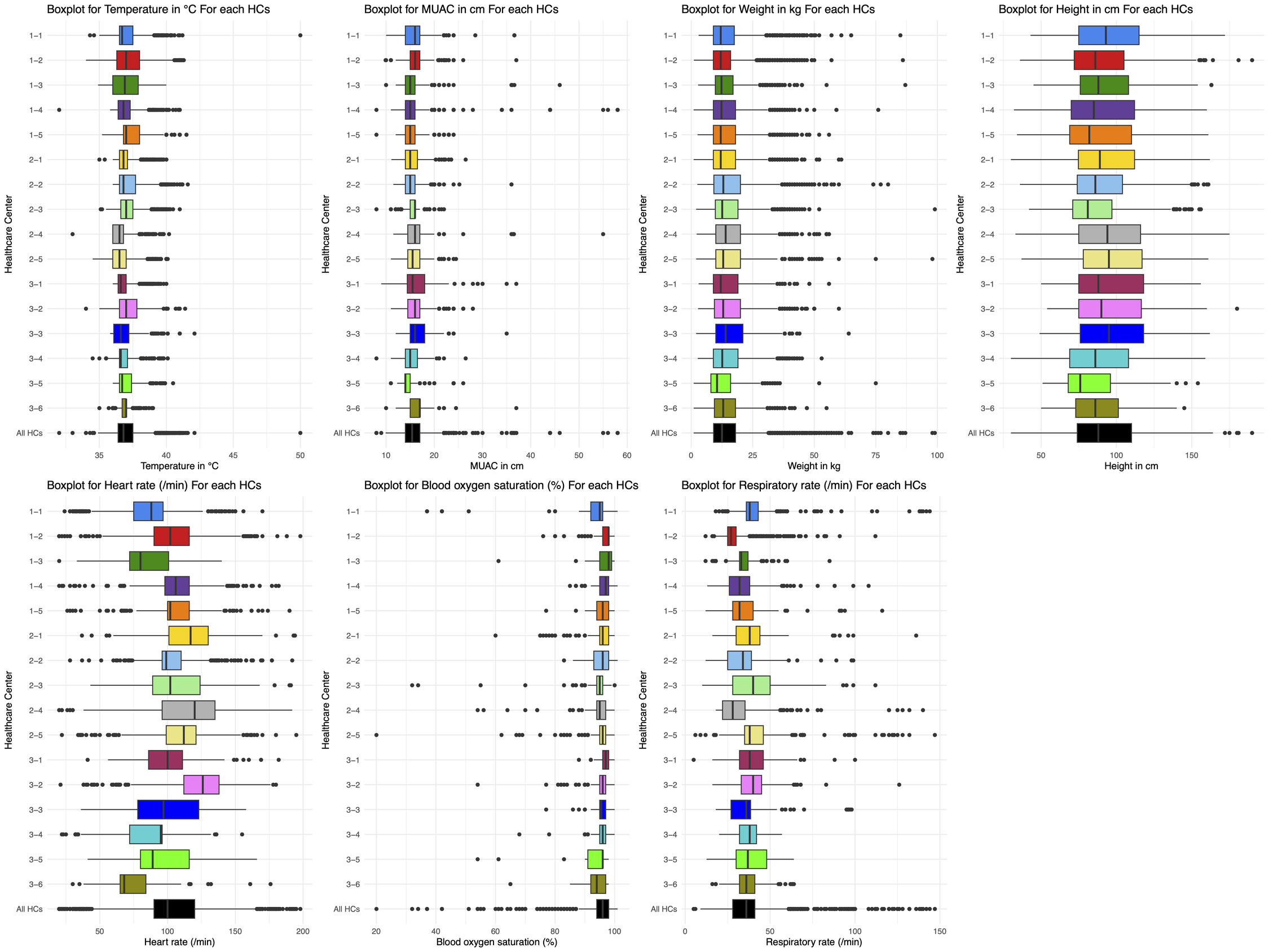

Supplement: S3 Fig — MUAC = mid-upper arm circumference. (TIF) [file pone.0318284.s004.tif]

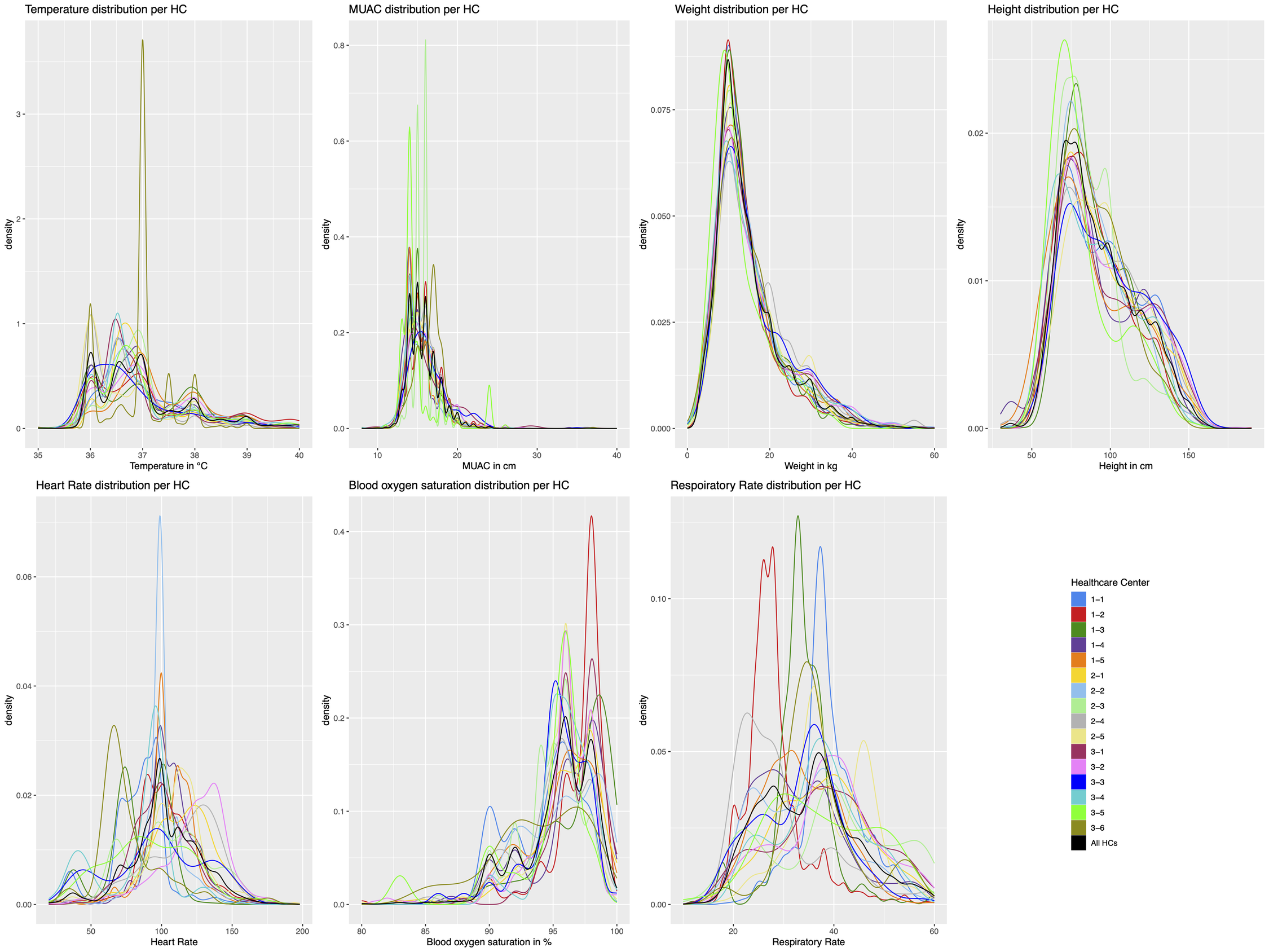

Supplement: S4 Fig — MUAC = mid-upper arm circumference. (TIF) [file pone.0318284.s005.tif]

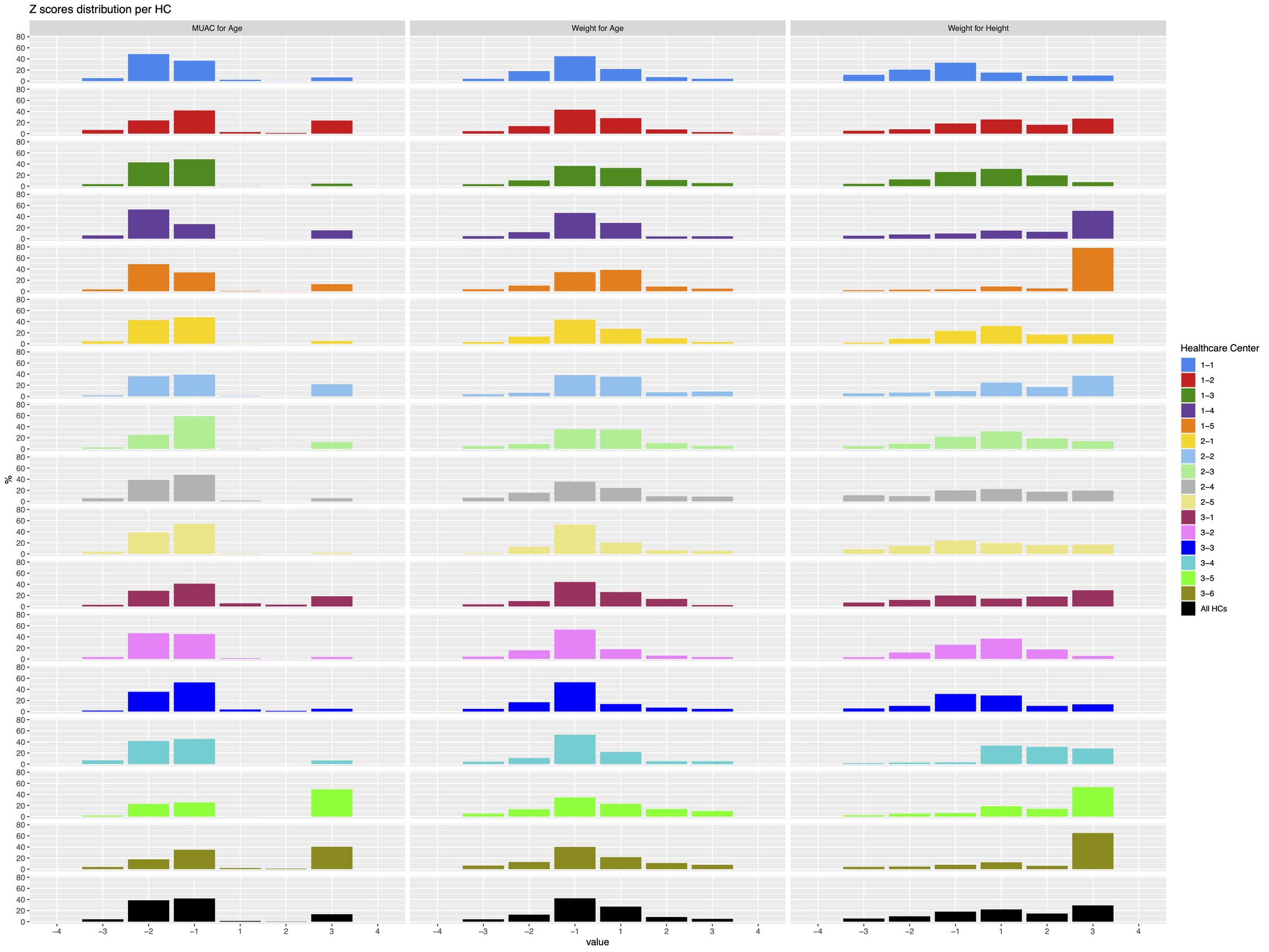

Supplement: S5 Fig — The analysis of Z-scores was conducted exclusively on data from children aged 2 months to 5 years, in accordance with the WHO Child Growth Standards. MUAC = mid-upper arm circumference. (TIF) [file pone.0318284.s006.tif]
