# Supplementary material for: Identifying clinical skill gaps of healthcare workers using a digital clinical decision support algorithm during outpatient pediatric consultations in primary health centers in Rwanda
Source: PLoS One. 2025 Jun 3;20(6):e0318284. doi: 10.1371/journal.pone.0318284 (PMC12132983; doi:10.1371/journal.pone.0318284)
Supplement: S1 Table — MUAC = mid-upper arm circumference; SpO2 = Blood oxygen saturation; HR = heart rate; RR = respiratory rate. (DOCX) [file pone.0318284.s003.docx]

| Healthcare Centers | Temperature | | MUAC | | Weight | | Height | | SpO_2_ | | HR | | RR | |
| --- | --- | --- | --- | --- | --- | --- | --- | --- | --- | --- | --- | --- | --- | --- |
|  | % | n/N | % | n/N | % | n/N | % | n/N | % | n/N | % | n/N | % | n/N |
| All HCs | 0,3 | 70/20052 | 11,6 | 2180/18752 | 0,3 | 58/20010 | 12,4 | 2324/18754 | 6,5 | 242/3724 | 18,4 | 1982/10757 | 13,2 | 1465/11090 |
| HC 1-1 | 0,1 | 2/2189 | 3,1 | 64/2092 | 0 | 0/2189 | 0,1 | 3/2092 | 1,7 | 12/695 | 6,2 | 83/1344 | 4,4 | 60/1349 |
| HC 1-2 | 0 | 0/2862 | 14,5 | 385/2656 | 0,6 | 17/2848 | 10,7 | 284/2657 | 13,8 | 36/261 | 26 | 440/1690 | 15,4 | 261/1699 |
| HC 1-3 | 0,3 | 6/2183 | 3,3 | 68/2050 | 0 | 1/2177 | 1 | 21/2050 | 39,7 | 60/151 | 57,3 | 481/839 | 52,5 | 449/856 |
| HC 1-4 | 0,7 | 11/1568 | 14,3 | 206/1445 | 0,2 | 3/1566 | 19 | 274/1445 | 6,4 | 14/219 | 11,2 | 114/1018 | 11,7 | 121/1035 |
| HC 1-5 | 0,3 | 3/1159 | 9,5 | 101/1063 | 0,8 | 9/1150 | 54,6 | 580/1063 | 3,1 | 6/192 | 1,1 | 8/716 | 2,1 | 15/717 |
| HC 2-1 | 0,1 | 1/898 | 4,1 | 35/847 | 0,2 | 2/898 | 2,8 | 24/848 | 6,7 | 19/285 | 4,6 | 26/571 | 2,1 | 12/574 |
| HC 2-2 | 0,1 | 1/1386 | 21,8 | 280/1282 | 0,1 | 2/1386 | 27 | 346/1282 | 2,2 | 4/183 | 1,5 | 10/683 | 0,4 | 3/685 |
| HC 2-3 | 0,1 | 1/1342 | 25,3 | 3241283 | 0,4 | 6/1337 | 19,7 | 253/1283 | 0,2 | 1/407 | 2,6 | 21/800 | 2,2 | 18/801 |
| HC 2-4 | 1 | 14/1456 | 15,9 | 212/1332 | 0,1 | 1/1455 | 3,2 | 43/1332 | 1,7 | 3/175 | 3 | 18/608 | 0,8 | 5/628 |
| HC 2-5 | 0,2 | 3/1327 | 1,4 | 18/1270 | 0,1 | 1/1327 | 0,9 | 12/1270 | 1,6 | 7/435 | 28 | 176/629 | 2 | 16/805 |
| HC 3-1 | 0,2 | 1/558 | 13,7 | 69/504 | 0,2 | 1/558 | 8,1 | 41/504 | 8,6 | 11/128 | 14,2 | 37/260 | 5,3 | 14/262 |
| HC 3-2 | 0 | 0/1030 | 2 | 19/955 | 0,1 | 1/1030 | 0 | 0/955 | 0 | 0/290 | 0 | 0/521 | 0,4 | 2/522 |
| HC 3-3 | 0,2 | 1/409 | 4,2 | 16/383 | 0 | 0/412 | 1 | 4/395 | 5,2 | 3/58 | 0 | 0/193 | 0 | 0/195 |
| HC 3-4 | 0,5 | 3/566 | 3,7 | 20/541 | 0,4 | 2/564 | 2,6 | 14/541 | 9,9 | 12/121 | 6 | 13/218 | 1,3 | 4/297 |
| HC 3-5 | 2,6 | 13/500 | 54,1 | 242/447 | 1,2 | 6/495 | 32,9 | 147/447 | 64,8 | 35/54 | 88,8 | 247/278 | 78,9 | 220/279 |
| HC 3-6 | 1,6 | 10/619 | 20,6 | 121/586 | 0,2 | 1/618 | 47,4 | 278/586 | 29,7 | 19/64 | 82,5 | 296/359 | 8,5 | 252/368 |
