# Supplementary material for: Identifying clinical skill gaps of healthcare workers using a digital clinical decision support algorithm during outpatient pediatric consultations in primary health centers in Rwanda
Source: PLoS One. 2025 Jun 3;20(6):e0318284. doi: 10.1371/journal.pone.0318284 (PMC12132983; doi:10.1371/journal.pone.0318284)
Supplement: S2 Table — (DOCX) [file pone.0318284.s007.docx]

| Question type | Question | Possible answer(s) |
| --- | --- | --- |
| **General** | | |
| Single Choice Question | Date | JJ/MM/YYYY |
| Single Choice Question | HC | HC 1-1 / HC 1-2 / HC 1-3 / HC 1-4 / HC 1-5 / HC 2-1 / HC 2-2 / HC 2-3 / HC 2-4 / HC 2-5 / HC 3-1 / HC 3-2 / HC 3-3 / HC 3-4 / HC 3-5 / HC 3-6 |
| Multiple Choice Question | Measures made before the consultation (At the reception) | T° / MUAC / Weight / Height / Saturation / HR / RR |
| Multiple Choice Question | Measures made during the consultation (by the HW) | T° / MUAC / Weight / Height / Saturation / HR / RR |
| Open-Ended Question | Conducting line? | … |
| Open-Ended Question | Remarks? | … |
| **Temperature** | | |
| Single Choice Question | T° Assessment | Assessed / Skipped |
| Open-Ended Question | If skipped, why? | … |
| Single Choice Question | T° Quality of Assessment | Sufficient / Insufficient |
| Open-Ended Question | If insufficient, why? | … |
| Open-Ended Question | Extra remark about T°? | … |
| **MUAC** | | |
| Single Choice Question | MUAC Assessment | Assessed / Skipped |
| Open-Ended Question | If skipped, why? | … |
| Single Choice Question | MUAC Quality of Assessment | Sufficient / Insufficient |
| Open-Ended Question | If insufficient, why? | … |
| Open-Ended Question | Extra remark about MUAC? | … |
| **Weight** | | |
| Single Choice Question | Weight Assessment | Assessed / Skipped |
| Open-Ended Question | If skipped, why? | … |
| Single Choice Question | Weight Quality of Assessment | Sufficient / Insufficient |
| Open-Ended Question | If insufficient, why? | … |
| Open-Ended Question | Extra remark about weight? | … |
| **Height** | | |
| Single Choice Question | Height Assessment | Assessed / Skipped |
| Open-Ended Question | If skipped, why? | … |
| Single Choice Question | Height Quality of Assessment | Sufficient / Insufficient |
| Open-Ended Question | If insufficient, why? | … |
| Open-Ended Question | Extra remark about height? | … |
| **Respiratory rate (RR)** | | |
| Single Choice Question | RR Assessment | Assessed / Skipped |
| Open-Ended Question | If skipped, why? | … |
| Single Choice Question | RR Quality of Assessment | Sufficient / Insufficient |
| Open-Ended Question | If insufficient, why? | … |
| Open-Ended Question | Extra remark about RR? | … |
| **Blood oxygen saturation (SpO_2_)** | | |
| Single Choice Question | SpO_2_ Assessment | Assessed / Skipped |
| Open-Ended Question | If skipped, why? | … |
| Single Choice Question | SpO_2_ Quality of Assessment | Sufficient / Insufficient |
| Open-Ended Question | If insufficient, why? | … |
| Open-Ended Question | Extra remark about SpO_2_? | … |
| **Heart rate (HR)** | | |
| Single Choice Question | HR Assessment | Assessed / Skipped |
| Open-Ended Question | If skipped, why? | … |
| Single Choice Question | HR Quality of Assessment | Sufficient / Insufficient |
| Open-Ended Question | If insufficient, why? | … |
| Open-Ended Question | Extra remark about HR? | … |
| **Extra** | | |
| Open-Ended Question | Remark about other sign or symptoms | … |
